# Trend analysis and projection of gastric cancer burden linked to high sodium intake in China, Japan, Republic of Korea, and Mongolia (1990–2021): A comprehensive assessment based on the 2021 global burden of disease study

**DOI:** 10.1371/journal.pone.0338030

**Published:** 2025-12-04

**Authors:** Xiaohuang Yang, Shaoxing Chen, Canmei Zhong, Yadong Lai, Fenglin Chen

**Affiliations:** 1 Department of Gastroenterology and Fujian Institute of Digestive Disease, Fujian Medical University Union Hospital, Fuzhou, Fujian, China; 2 Department of Gastroenterology, Zhangzhou Affiliated Hospital of Fujian Medical University, Zhangzhou, Fujian, China; 3 Department of Radiation Oncology, Zhangzhou Affiliated Hospital of Fujian Medical University, Zhangzhou, Fujian, China; Lithuanian University of Health Sciences, LITHUANIA

## Abstract

**Background:**

High sodium intake (HSI) is one of the risk factors for gastric cancer. China, Japan, Republic of Korea, and Mongolia are among the countries with the highest gastric cancer incidence worldwide. This study aimed to assess the burden of gastric cancer linked to HSI in these four countries from 1990 to 2021, and to project future trends.

**Methods:**

The 2021 Global Burden of Disease (GBD) database was used to analyze trends in HSI-related gastric cancer burden and differences by age and sex in the four countries. Trend changes were evaluated using Joinpoint regression. Decomposition analysis was conducted to assess the relative contributions of demographic aging, population growth, and epidemiological shifts. Future burden was forecast using a Bayesian age–period–cohort (BAPC) model.

**Results:**

In 2021, the number of gastric cancer deaths in China linked to HSI was 36,958 (95% UI: 0–183,972), with 883,435 DALYs (95% UI: 0–4,461,211). The age-standardized mortality rate (ASMR) was 1.78 (95% UI: 0–8.81) per 100,000, and the age-standardized DALY rate (ASDR) was 41.49 (95% UI: 0–208.59) per 100,000. Across the four countries, the burden of gastric cancer due to HSI was greater in males and elderly groups. Trends in ASMR and ASDR showed consistent declines in all four countries according to Joinpoint analysis. Decomposition analysis demonstrated that epidemiological shifts contributed to easing the burden. The BAPC model projected that the ASMR and ASDR of HSI-related gastric cancer would decrease over the upcoming 15 years in the four countries.

**Conclusions:**

During 1990–2021, ASMR and ASDR for HSI-related gastric cancer declined in China, Japan, Republic of Korea, and Mongolia, yet differences by sex and age remained. Policymakers need to formulate targeted public health measures in light of these differences.

## Introduction

Gastric cancer is a common malignant tumor of the digestive tract. According to GLOBOCAN 2022, it ranks fifth in both incidence and mortality among all cancers, with an estimated 968,350 new cases and 659,853 deaths worldwide [1]. Substantial geographical variation exists, with incidence and mortality generally lower in high-income countries [2]. U.S. cancer statistics project 30,300 new gastric cancer cases and 10,780 deaths in 2025, with both incidence and mortality falling outside the top ten cancers [3]. In 2021, East Asia had the highest global age-standardized incidence rate (ASIR) of gastric cancer, especially in China, Mongolia, Japan, and Republic of Korea [2].

Gastric cancer arises from a complex interplay of factors, including Helicobacter pylori (HP) infection, dietary and lifestyle patterns, and genetic susceptibility [4,5]. Lifestyle factors, particularly smoking and excessive salt intake, contribute to the development and progression of gastric cancer [6]. High sodium intake (HSI) increases the likelihood of HP colonization, thereby elevating the risk of gastric cancer [7]. Furthermore, excessive salt intake impairs the gastric mucosa, which further boosts DNA synthesis and cell proliferation to accelerate gastric cancer progression [8]. Evidence from multiple studies consistently indicates that HSI confer a substantially greater risk of gastric cancer compared with lower salt intake [9–11].

As countries with high gastric cancer incidence in East Asia, China, Japan, Republic of Korea, and Mongolia have a close association between their dietary habits, food types, and gastric cancer. According to the China National Nutrition and Health Survey, the average daily salt intake of Chinese residents was 10.5 g in 2012, which was substantially higher than the amount recommended by the World Health Organization (WHO) [12]. Additionally, a study reported that Chinese residents’ preference for pickled vegetables was positively correlated with gastric cancer risk (hazard ratio [HR] = 1.17; 95% uncertainty interval [UI] = 1.00–1.37; P = 0.039) [13]. Research has shown that in 2019, Japanese men consumed an average of 10.9 g of salt per day, and Japanese women 9.3 g per day [14]. Traditional Japanese dietary practices rely heavily on salt for making foods like salted fish, pickled vegetables, and soy sauce. A study conducted in Japan also found that HSI were associated with a further elevated risk of gastric cancer in the Japanese population [15]. According to the 2017 Korean Health Statistics, the average daily sodium intake in Korea was 3,477.2 mg, substantially exceeding the WHO’s recommended limit of 2,000 mg/day [16]. Kimchi, a traditional Korean fermented vegetable dish, has been associated with an increased risk of gastric cancer [17]. Additionally, a study indicated that the per capita daily salt intake in Mongolia was 11.1 g in 2011, which was also well above the WHO’s recommended level [18]. While global gastric cancer incidence has declined due to advances in healthcare and the enforcement of relevant policies [19], these four high-incidence countries have unique dietary habits that are linked to gastric cancer development and progression. Conducting further in-depth epidemiological research on HSI-related gastric cancer in these four nations is especially critical, which can offer scientific evidence to guide the development of gastric cancer prevention and control strategies.

This study leveraged the 2021 Global Burden of Disease (GBD) database to conduct a comprehensive analysis of the gastric cancer burden caused by HSI in China, Japan, Republic of Korea, and Mongolia. It examined temporal trends in HSI-associated gastric cancer and disparities by sex and age, and predicted disease burden trends for the next 15 years, which can serve as a reference to guide the development of targeted public health policies.

## Methods

### Data source

The 2021 GBD database reports estimated epidemiological data for 371 diseases and injuries across 204 countries and territories over the period 1990–2021 [20,21]. GBD data are derived from sources such as vital registration systems, population censuses, household surveys, disease-specific registries, clinical datasets, and police records [22,23]. In this study, data on HSI-related gastric cancer burden and 95% UIs for China, Japan, Republic of Korea, and Mongolia (1990–2021) were downloaded using the Global Health Data Exchange (GHDx) query tool (https://vizhub.healthdata.org/gbd-results/). Within the GBD framework, UIs for all metrics were generated using 1,000 draws, with the 95% UI defined by the 2.5th and 97.5th percentiles of the distribution [20].

### Definitions

According to the International Classification of Diseases, gastric cancer is defined as all diagnoses coded 151–151.9 in ICD-9 and C16–C16.9 in ICD-10. The WHO recommends that adults consume less than 5 g of salt, equivalent to 2 g of sodium, per day [24]. A high-sodium diet is therefore defined as a dietary pattern in which sodium intake exceeds the WHO recommendation.

### Socio-demographic index (SDI)

The SDI is a composite indicator of socioeconomic development, calculated from per capita income, mean years of schooling, and total fertility rate [25]. Values range from 0 to 1, with higher scores reflecting more advanced social development. We applied Spearman’s correlation test to examine the associations between SDI values and the Age-standardized mortality rate (ASMR) and Age-standardized DALYs (disability-adjusted life years) rate (ASDR) of gastric cancer linked to HSI in the four countries from 1990 to 2021. Locally Estimated Scatterplot Smoothing (LOESS) was used to generate fitted curves to better visualize these relationships. Spearman’s correlation coefficients (ρ) range from −1–1, with positive values indicating positive correlation, negative values indicating inverse correlation, and larger absolute values reflecting stronger associations [26].

### Joinpoint regression analysis

Joinpoint regression analysis was employed to evaluate temporal trends in HSI-associated gastric cancer [27]. This method splits data into multiple phases, enabling further identification and quantification of change points in the data, and calculates the annual percentage change (APC) and average annual percentage change (AAPC) [28]. The AAPC provides an estimate of the disease’s trend from 1990 to 2021. A 95% confidence interval (CI) for AAPC with a lower bound greater than 0 indicates an upward trend, whereas an upper bound less than 0 indicates a downward trend.

### Age–period–cohort analysis

The Age–Period–Cohort model is a widely used statistical approach in epidemiology and demography [29]. This model simultaneously accounts for the effects of age, period, and cohort on disease patterns. We applied the Age–Period–Cohort web tool developed by the U.S. National Cancer Institute (https://analysistools.cancer.gov/apc/) to evaluate the influence of these three factors on gastric cancer mortality linked to HSI in China, Japan, Republic of Korea, and Mongolia [29]. As the GBD 2021 database does not provide estimates for individuals younger than 25 years, our analysis included 15 age groups (in 5-year intervals, ranging from 25–29 years to ≥95 years), 6 consecutive 5-year periods (1992–1996 to 2017–2021), and 20 birth cohorts (1892–1996). The period 2002–2006 and the 1942 birth cohort were selected as reference categories. Net and local drifts were used to evaluate temporal changes in mortality, with net drifts >0 indicating an upward trend.

### Decomposition analysis

Decomposition analysis is a method used to investigate changes in specific indicators, which decomposes changes in an indicator into multiple factors to further assess the contribution of each factor to the overall change [30,31]. In this study, decomposition analyses were conducted stratified by sex for each country to estimate the contributions of population aging, epidemiological changes, and population growth to changes in deaths and DALYs from gastric cancer linked to HSI.

### Predictive analysis

A Bayesian age-period-cohort (BAPC) model was employed to forecast the trajectory of gastric cancer burden due to HSI in the four countries up to 2036 [32]. This model accounts for age, period, and cohort effects and leverages Integrated Nested Laplace Approximations (INLA) to estimate their influence on future disease trends [33].

### Data visualization

All analyses were performed using R software (version 4.4.2) and Joinpoint Software (version 5.2.0), with results visualized to enhance interpretability. A P-value of less than 0.05 indicates statistical significance.

### Ethical considerations

This study conducted a secondary analysis using the 2021 GBD database, for which ethical approval and informed consent were not required.

## Results

### Comparison of gastric cancer burden linked to HSI, 1990–2021

**Fig 1:**
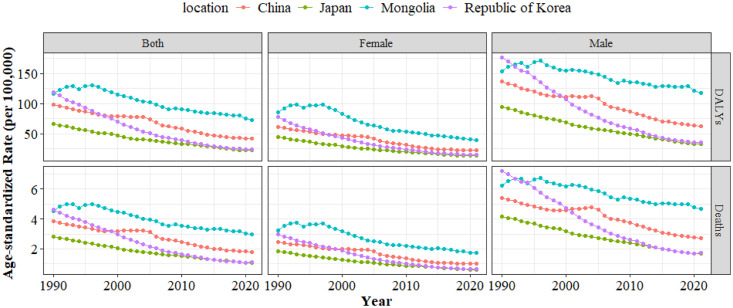
Trends in ASMR and ASDR of gastric cancer linked to HSI from 1990 to 2021. ASMR, Age-standardized mortality rate; ASDR, Age-standardized DALYs (disability-adjusted life years) rate; HSI, High Sodium Intake.

Between 1990 and 2021, deaths from gastric cancer linked to HSI increased in China from 31,208 (95%UI: 0–152,476) to 36,958 (95% UI: 0–183,972), representing an 18.4% rise. In Japan, deaths rose slightly from 4,680 (95% UI: 0–23,011) to 4,801 (95% UI: 0–24,191), a 2.6% increase. Mongolia experienced an increase from 47 (95% UI: 0–230) to 65 (95% UI: 0–340), corresponding to a 38.3% rise. Conversely, in Republic of Korea, deaths decreased from 1,348 (95% UI: 0–6,571) to 1,029 (95% UI: 0–5,084), a 23.7% decline. From 1990 to 2021, DALYs from gastric cancer linked to HSI decreased by 1.05%, 31.00%, and 45.87% in China, Japan, and Republic of Korea, respectively, whereas Mongolia experienced a 46.80% increase (Table 1). Over the same period, ASMR and ASDR declined in all four countries. Republic of Korea exhibited the most substantial reductions, with AAPCs of −4.53 (95% CI: −4.8 to −4.26) for ASMR and −5.04 (95% CI: −5.29 to −4.78) for ASDR (S1 Table, Fig 1). Before 2000, ASMR and ASDR in Republic of Korea were higher than those in China; after 2000, these rates in China exceeded those in Republic of Korea (Fig 1).

**Table 1:** All-age cases and ASMR, ASDR in 1990 and 2021 for gastric cancer linked to HSI.

Location	Measure	Sex	1990		2021	
			All-ages cases	Age-standardized rates per 100,000 people	All-ages cases	Age-standardized rates per 100,000 people
			n (95% UI)	n (95% UI)	n (95% UI)	n (95% UI)
China	Deaths	Male	21063 (0-103279)	5.42 (0-26.71)	26171 (0-136689)	2.71 (0-14.07)
China	Deaths	Female	10146 (0-49845)	2.48 (0-12.12)	10786 (0-55030)	0.99 (0-5.06)
China	Deaths	Both	31208 (0-152476)	3.85 (0-18.79)	36958 (0-183972)	1.78 (0-8.81)
Japan	Deaths	Male	2931 (0-14379)	4.18 (0-20.49)	3046 (0-15264)	1.68 (0-8.45)
Japan	Deaths	Female	1749 (0-8754)	1.85 (0-9.25)	1755 (0-9163)	0.63 (0-3.22)
Japan	Deaths	Both	4680 (0-23011)	2.81 (0-13.86)	4801 (0-24191)	1.09 (0-5.46)
Republic of Korea	Deaths	Male	849 (0-4213)	7.19 (0-35.74)	669 (0-3359)	1.71 (0-8.52)
Republic of Korea	Deaths	Female	500 (0-2445)	3 (0-14.66)	360 (0-1758)	0.68 (0-3.3)
Republic of Korea	Deaths	Both	1348 (0-6571)	4.61 (0-22.14)	1029 (0-5084)	1.11 (0-5.5)
Mongolia	Deaths	Male	28 (0-139)	6.23 (0-30.61)	45 (0-240)	4.66 (0-24.65)
Mongolia	Deaths	Female	18 (0-95)	3.24 (0-16.71)	20 (0-114)	1.72 (0-9.7)
Mongolia	Deaths	Both	47 (0-230)	4.52 (0-22.13)	65 (0-340)	2.96 (0-15.68)
China	DALYs	Male	615930 (0-3026942)	136.67 (0-668.68)	643008 (0-3367599)	62.16 (0-323.76)
China	DALYs	Female	276885 (0-1368559)	61.39 (0-302.16)	240427 (0-1225234)	22.15 (0-112.91)
China	DALYs	Both	892815 (0-4340140)	98.4 (0-478.5)	883435 (0-4461211)	41.46 (0-208.59)
Japan	DALYs	Male	70972 (0-350693)	93.97 (0-463.1)	51458 (0-257834)	33.08 (0-164.98)
Japan	DALYs	Female	39953 (0-197553)	43.97 (0-217.15)	25079 (0-129092)	13.04 (0-66.32)
Japan	DALYs	Both	110926 (0-544309)	65.73 (0-322.56)	76537 (0-383664)	22.26 (0-111.56)
Republic of Korea	DALYs	Male	25588 (0-125362)	176.68 (0-876.88)	14752 (0-74328)	35.2 (0-176.51)
Republic of Korea	DALYs	Female	14607 (0-71877)	77.39 (0-378.65)	7005 (0-34610)	15.09 (0-74.72)
Republic of Korea	DALYs	Both	40195 (0-196098)	118.73 (0-580.64)	21757 (0-106998)	24.08 (0-117.95)
Mongolia	DALYs	Male	786 (0-3879)	154.24 (0-759.77)	1373 (0-7267)	117.58 (0-623.39)
Mongolia	DALYs	Female	511 (0-2651)	85.42 (0-445.31)	531 (0-3017)	39 (0-222.18)
Mongolia	DALYs	Both	1297 (0-6470)	116.56 (0-576.28)	1904 (0-9843)	73.19 (0-380.58)

ASMR, Age-standardized mortality rate; ASDR, Age-standardized DALYs (disability-adjusted life years) rate; HSI, High Sodium Intake.

### Gender and age disparities in gastric cancer burden linked to HSI from 1990 to 2021

**Fig 2:**
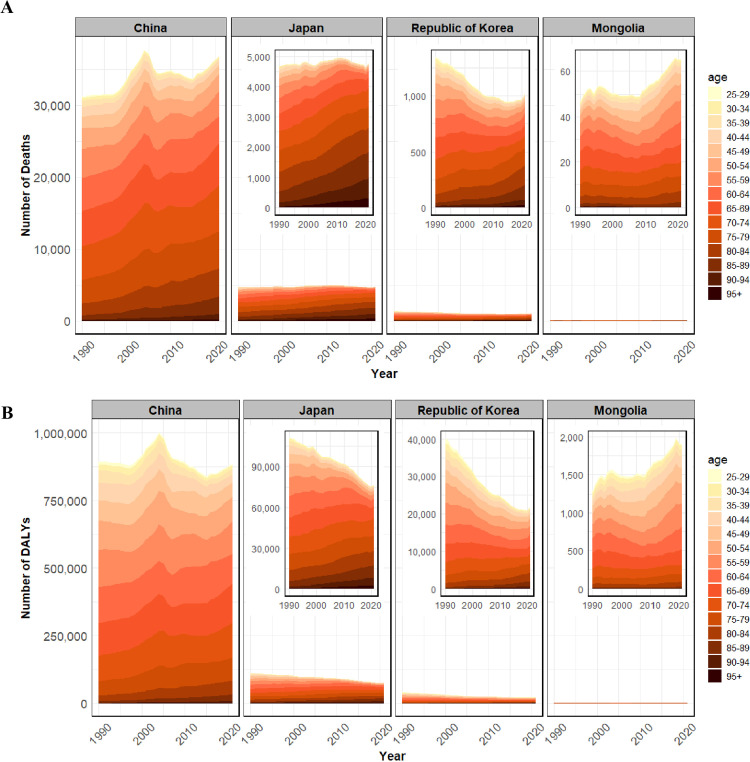
Trends for the age-specific absolute burden of gastric cancer linked to HSI,1990 - 2021, both sexes. **(A)** Number of deaths. **(B)**Number of DALYs. HSI, High Sodium Intake; DALYs, disability-adjusted life years.

In China, gastric cancer deaths linked to HSI increased from 21,063 (95% UI: 0–103,279) in men and 10,146 (95% UI: 0–49,845) in women in 1990, with a male-to-female ratio of 2.08, rising to 2.43 in 2021. Similarly, the DALY ratio increased from 2.22 in 1990 to 2.67 in 2021, indicating a widening gender gap (Table 1). In contrast, Japan showed a less pronounced expansion, with male-to-female ratios for deaths and DALYs of 1.68 and 1.78 in 1990, compared with 1.73 and 2.05 in 2021 (Table 1). Throughout most of the study period, both male and female ASMR and ASDR for gastric cancer linked to HSI were higher in Mongolia and lower in Japan compared with other countries (Fig 1). Korea exhibited the most pronounced decline in both indicators for men and women between 1990 and 2021 (Fig 1). Across all four countries, men consistently had higher ASMR and ASDR than women (Fig 1). In 2021, gastric cancer mortality and DALY rates linked to HSI increased with age in China, Japan, Republic of Korea, and Mongolia (S1 Fig). From 1990 to 2021, absolute deaths and DALYs rose in older age groups across all four countries (Fig 2). In contrast, declines were observed in individuals younger than 45 years in Republic of Korea (Fig 2). Stratified by sex, both men and women showed similar upward trends with advancing age (S2 Fig). These findings highlight the persistent burden among older populations.

### Temporal trends in age-standardized rates (ASRs) of gastric cancer linked to HSI, 1990–2021

**Fig 3:**
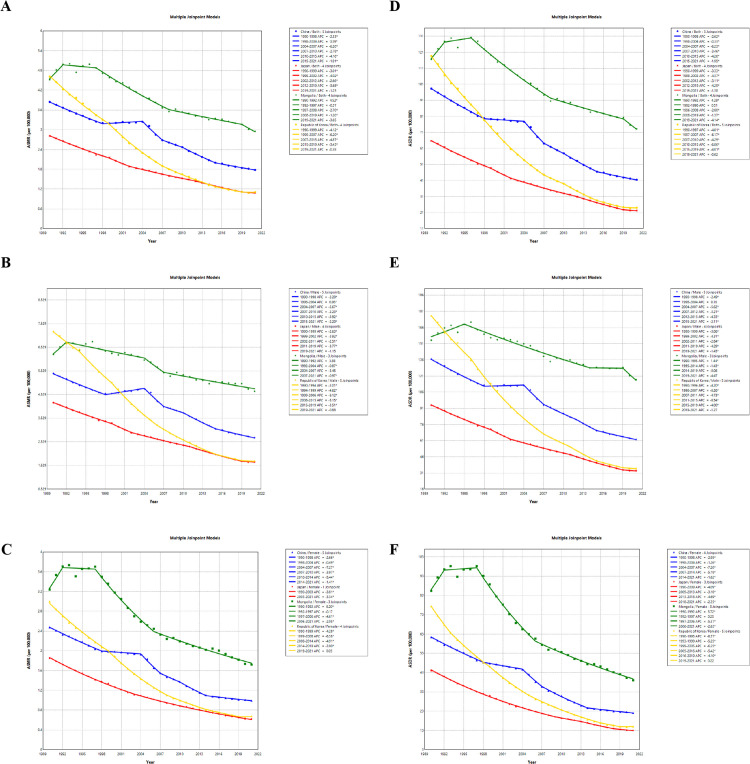
Joinpoint regression of ASMR and ASDR of gastric cancer linked to HSI across four countries, 1990–2021. **(A)** ASMR among both sexes. **(B)** ASMR among Males. **(C)** ASMR among Females. **(D)** ASDR among both sexes. **(E)** ASDR among Males. **(F)** ASDR among Females. “*” indicates p < 0.05. ASR, Age-standardized rate; ASMR, Age-standardized mortality rate; ASDR, Age-standardized DALYs (disability-adjusted life years) rate; HSI, High Sodium Intake.

Joinpoint regression showed that China’s ASMR of gastric cancer linked to HSI experienced six period-specific changes, with an overall declining trend. A modest increase was observed during 1998–2004 (APC = 0.39, 95% CI: 0.15–0.63), while the most recent period (2015–2021) showed a significant decrease (APC = –1.91, 95% CI: –2.09 to –1.73). Both sexes exhibited downward trajectories, with AAPCs of –2.22 (95% CI: –2.44 to –2.01) for men and –2.91 (95% CI: –3.12 to –2.70) for women (Fig 3A–C; S1 Table). Between 1990 and 2021, the ASMR and ASDR of HSI-linked gastric cancer in both sexes, men and women in Japan and Republic of Korea exhibited a relatively smooth downward trend, with no significant fluctuations observed in any period (Fig 3A–F). In contrast, the ASMR and ASDR of HSI-associated gastric cancer in both sexes, men and women in Mongolia showed a trend of first increasing and then decreasing (Fig 3A–F).

### Age–period–cohort analysis of gastric cancer mortality linked to HSI

**Fig 4:**
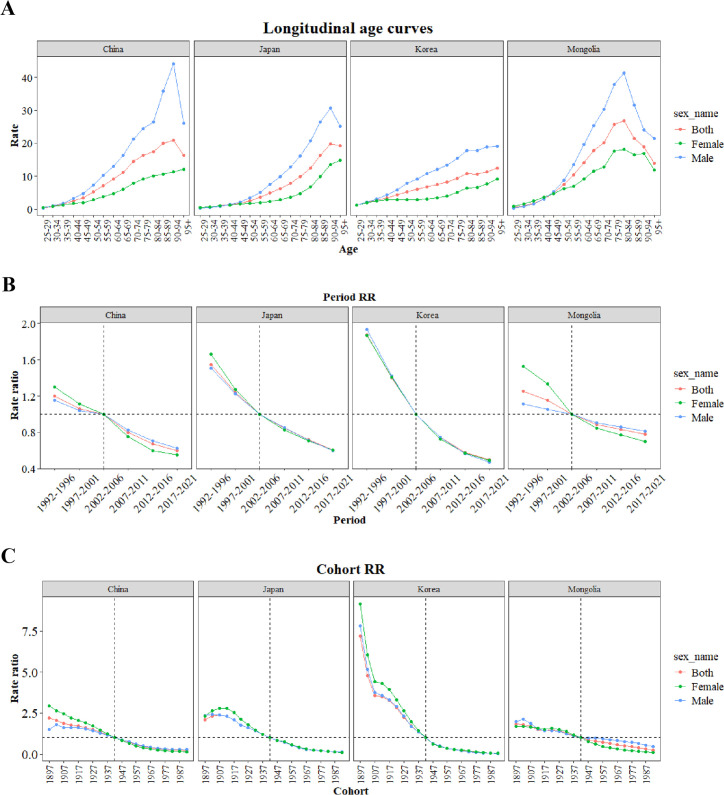
Age–specific, period, and cohort effects on gastric cancer mortality linked to HSI. **(A)** Age effect. **(B)** Period effect. **(C)** Cohort effect. HSI, High Sodium Intake.

**Fig 5:**
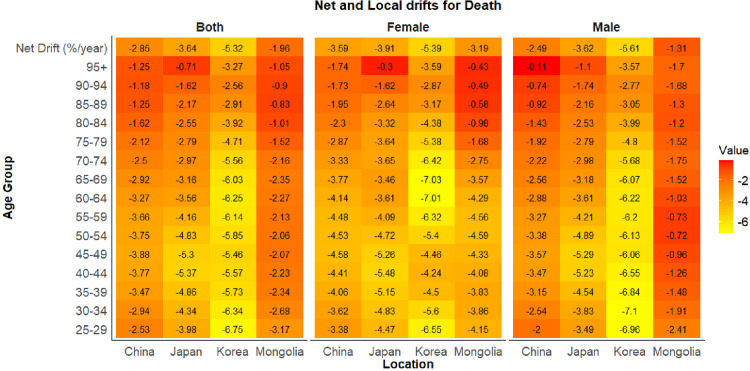
Net and Local drifts for Gastric cancer mortality rate linked to HSI. HSI, High Sodium Intake.

According to longitudinal age curves, age-specific death rates of gastric cancer linked to HSI increased with advancing age in China, Japan, Republic of Korea, and Mongolia. In all four countries, men consistently exhibited higher rates than women (Fig 4A). Period effects for gastric cancer mortality linked to HSI exhibited similar patterns in China and Mongolia. Prior to 2002–2006, relative risks (RRs) were higher in women than in men and the overall population. After 2002–2006, RRs in men and the total population exceeded those in women. In Japan and Republic of Korea, RRs consistently declined, with Republic of Korea showing a more pronounced decrease (Fig 4B). Regarding cohort effects, RRs of gastric cancer mortality linked to HSI in Republic of Korea differed markedly from those in the other three countries. The RRs declined rapidly for cohorts born between 1897 and 1907 and decreased more gradually for cohorts born between 1907 and 1942. In Japan, RRs peaked for the 1902–1911 birth cohort and then declined steadily (Fig 4C). The net drift results highlighted an overall decline in gastric cancer mortality linked to HSI across the four countries. In China, the local drift for the 25–29years group was smaller in absolute value than the net drift, indicating a weaker downward trend in this age group compared with the overall trend. For individuals aged 75 years and older, absolute local drifts in all four countries were smaller than the net drift, suggesting that the decline in older populations was less pronounced than the overall trend (Fig 5).

### Decomposition analysis of gastric cancer burden linked to HSI

**Fig 6:**
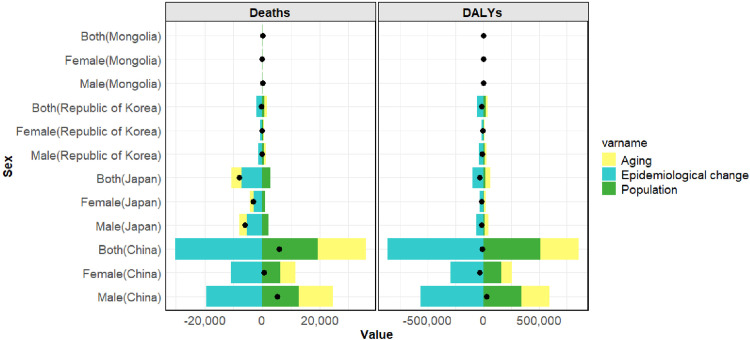
Decomposition of gastric cancer deaths and DALYs linked to HSI by gender. HSI, High Sodium Intake; DALYs, disability-adjusted life years.

Fig 6 presents the decomposition analysis of gastric cancer deaths and DALYs linked to HSI in China, Japan, Republic of Korea, and Mongolia from 1990 to 2021. Aging and population growth were the primary drivers of deaths in China, Republic of Korea, and Mongolia, whereas changes in epidemiology acted as a mitigating factor. Interestingly, population aging in Japan contributed to a reduction in deaths. Decomposition of DALYs showed that aging and population growth positively contributed to DALYs in all four countries, with population growth being the main factor in Mongolia. Epidemiological changes consistently mitigated DALYs across all countries (Fig 6, S3 Fig).

### Association between ASRs and SDI for gastric cancer linked to HSI

**Fig 7:**
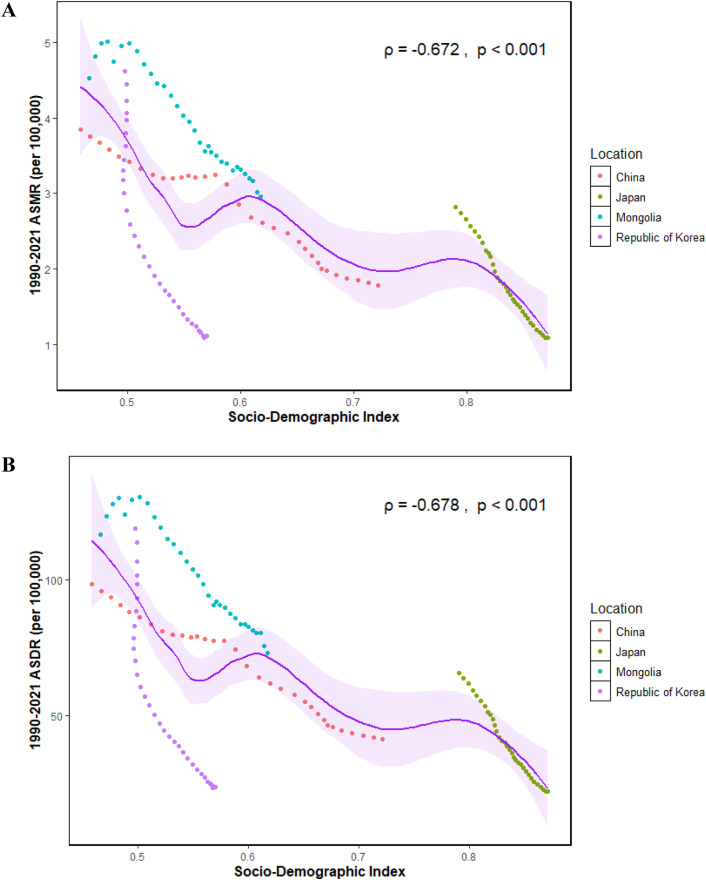
ASMR and ASDR of gastric cancer linked to HSI from 1990 to 2021 according to SDI. **(A)** ASMR. **(B)** ASDR. The purple line was an adaptive association fitted with adaptive Loess regression based on all data points. ASMR, Age-standardized mortality rate; ASDR, Age-standardized DALYs (disability-adjusted life years) rate; HSI, High Sodium Intake; SDI, Socio-Demographic Index.

The ASMR and ASDR of gastric cancer linked to HSI showed similar trends in relation to the SDI in China, Japan, Republic of Korea, and Mongolia (Fig 7A–B). With increasing SDI, ASMR and ASDR declined sharply in Japan and Republic of Korea, while China exhibited a more gradual decrease. In Mongolia, ASMR and ASDR initially increased and then decreased, peaking at an SDI of 0.5. Correlation analyses confirmed a significant negative association between SDI and gastric cancer burden linked to HSI, with ρ = –0.672 (P < 0.001) for ASMR and ρ = –0.678 (P < 0.001) for ASDR.

### Forecast of gastric cancer burden linked to HSI

**Fig 8:**
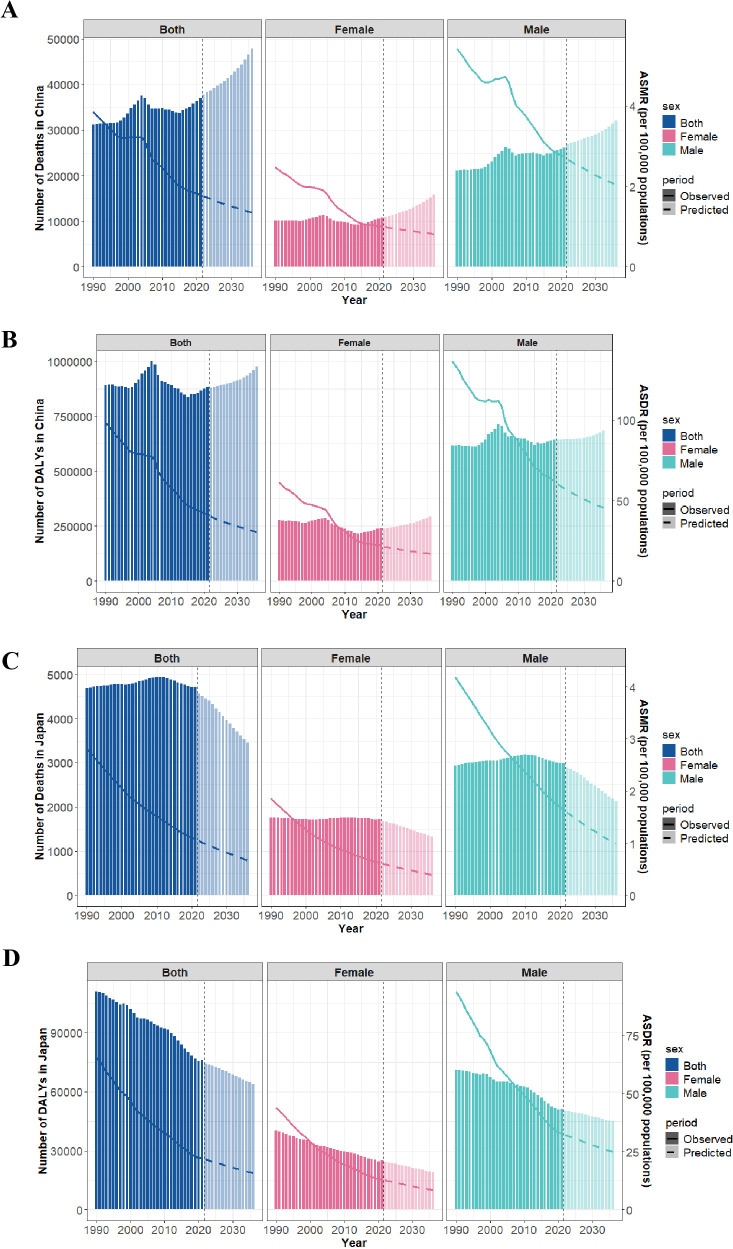
Projections of gastric cancer burden linked to HSI by gender through 2036. **(A)** ASMR and Number of deaths in China. **(B)** ASDR and Number of DALYs in China. **(C)** ASMR and Number of deaths in Japan. **(D)** ASDR and Number of DALYs in Japan. ASMR, Age-standardized mortality rate; ASDR, Age-standardized DALYs (disability-adjusted life years) rate; HSI, High Sodium Intake.

The BAPC model was used to project the burden of gastric cancer linked to HSI in China, Japan, Republic of Korea, and Mongolia through 2036. In China, both the ASMR and ASDR are expected to continue declining, with ASMR decreasing from 1.78 per 100,000 in 2021 to 1.35 per 100,000 in 2036, and ASDR declining from 41.46 per 100,000 in 2021 to 30.52 per 100,000 in 2036 (Fig 8A–B, S2 Table). Despite this, the absolute number of deaths and DALYs is projected to increase. Deaths are expected to rise from 36,958 in 2021–47,885 in 2036, representing a 29.57% increase, while DALYs are predicted to grow from 883,435–979,000, an increase of 10.82% (Fig 8A–B, S2 Table). The ASMR and ASDR of gastric cancer linked to HSI in Japan, Republic of Korea, and Mongolia are projected to decline over the next 15 years (Fig 8C–D, S4 Fig). The absolute disease burden varies across these countries. In Japan, deaths are expected to decrease from 4,801 in 2021–3,458 in 2036, a reduction of 27.97%, and DALYs are projected to decline from 76,537–63,939, a 19.70% reduction. In Republic of Korea, deaths are projected to decrease from 1,029–967, a 6.02% reduction, whereas DALYs are expected to rise from 21,757–26,403, an increase of 21.35%. In Mongolia, both deaths and DALYs are projected to increase, with deaths rising from 65 to 102, a 56.92% increase, and DALYs increasing from 1,904–2,423, a 27.26% increase (Fig 8C–D, S4 Fig, S3–S5 Tables). Sex-specific analysis indicates that ASMR and ASDR are projected to decline in both men and women in all four countries (Fig 8, S4 Fig).

## Discussion

Using data from the GBD 2021 study, we comprehensively analyzed the gastric cancer burden linked to HSI in China, Japan, Republic of Korea, and Mongolia. Despite overall reductions in ASMR and ASDR between 1990 and 2021, notable differences persist by sex, age, and country. These declines likely reflect advances in prevention and treatment strategies. However, considering the persistently high incidence of gastric cancer in these countries, the gastric cancer burden linked to HSI continues to represent a major public health challenge.

Our study revealed that the gastric cancer burden linked to HSI was consistently higher in men than in women across China, Japan, Republic of Korea, and Mongolia, indicating a marked sex disparity, in line with previous reports [34,35]. Smoking and alcohol consumption have been well established as major risk factors for gastric cancer [36,37]. Notably, men who smoke and drink tend to prefer salty foods [38,39], and the combination of multiple risk factors further accelerates gastric carcinogenesis. Additionally, due to social roles, men are more likely to dine out, thereby increasing their exposure to HSI. Studies have shown that men excrete greater amounts of sodium in urine (247 mmol/day) than women (218 mmol/day), indicating that men are subjected to higher sodium exposure within the internal environment, thereby facing a higher disease burden [40]. To address this sex disparity, policymakers should implement targeted interventions to reduce the gastric cancer burden linked to HSI in men. Health education programs aimed at increasing awareness of the harmful effects of HSI, particularly among men who smoke and consume alcohol, are essential, as these behaviors exert synergistic effects on gastric cancer risk. Furthermore, promoting the availability of low-salt options in restaurants and canteens may support healthier dietary choices. From a healthcare perspective, community-based systematic screening for gastric cancer should prioritize high-risk men, including those with high-sodium dietary habits or a history of smoking and drinking, to facilitate early detection and timely treatment.

We found that the ASMR and ASDR of HSI-linked gastric cancer were positively correlated with increasing age in China, Japan, Republic of Korea, and Mongolia, indicating that older adults are more susceptible to the development and progression of gastric cancer induced by HSI. A high-salt diet disrupts the protective layer of the gastric mucosa, leading to gastric mucosal damage and inflammatory responses, which in turn increase the risk of HP infection [15,41]. However, older adults exhibit impaired gastric mucosal repair capacity, making it more difficult for the gastric mucosa to recover after stimulation by high sodium. Additionally, age-related decline in immune function reduces the ability of the immune system to effectively clear HP, increasing the likelihood of progression to chronic gastritis, a key precursor to gastric carcinogenesis. Nutrients such as vitamin A, vitamin C, and folate, which are abundant in fruits and vegetables, have been shown to promote regeneration and repair of gastric mucosal cells [42,43]. Previous studies have reported a decline in fruit and vegetable consumption among older adults in all four countries, although to varying degrees [44–47]. This reduction may further increase the risk of gastric cancer linked to HSI in the elderly. Future public health policy development should prioritize the elderly, with a focus on promoting low-sodium diets. Through community talks and health promotion programs, older adults and their offspring should be informed about the risks of HSI and the value of low-sodium eating, supporting changes to daily diets such as less pickled food consumption and more fruit and vegetable intake. Health sectors could establish daily sodium intake guidelines aligned with the physiological requirements of older adults, with individualized modifications based on health status (e.g., hypertension, diabetes). For healthcare provision, regular gastroscopy and HP testing should be offered to older individuals, especially those who consume HSI. Governments and relevant bodies should drive the food industry to lower sodium levels in processed foods and enforce clear sodium labeling on packaging, enabling older adults to select low-sodium options more easily.

Joinpoint analysis identified declining trends in the ASMR and ASDR of HSI-related gastric cancer across China, Japan, Republic of Korea, and Mongolia from 1990 to 2021. In 2011, Mongolia’s Ministry of Health developed a national salt reduction strategy, with phased pilots in selected cities to lower population-wide sodium consumption [18]. The policy was formally implemented in 2020–2021, suggesting that Mongolia’s disease burden of HSI-associated gastric cancer may decline further in the coming years [48]. Furthermore, Mongolia established its National Cancer Council in 2014, which has enhanced efforts to prevent and manage malignant tumors, supporting a reduction in gastric cancer mortality [49]. Due to regional and dietary differences, the dietary habits in northern and southern China differ markedly, with northern populations consuming significantly higher amounts of salt. Since 2007, China has implemented effective salt-reduction policies, including standardized dietary guidelines emphasizing controlled salt and oil intake, as well as regulations requiring clear sodium labeling on packaged foods [50]. The enforcement of these policies has contributed to mitigating the burden of gastric cancer linked to HSI in China. Japan and Republic of Korea have similarly implemented salt-reduction policies to prevent a range of HSI-related diseases, including hypertension, cardiovascular disease, and gastric cancer [51–53]. As high-incidence and economically developed countries, both nations have established comprehensive gastric cancer screening programs, encompassing endoscopic screening and eradication of HP [54,55], which have effectively reduced gastric cancer incidence and mortality. The combined implementation of salt-reduction measures and targeted gastric cancer prevention strategies has contributed to a notable decline in ASMR and ASDR linked to HSI in these four countries from 1990 to 2021. Continued policy refinement and strengthened public education and health awareness are warranted to sustain and further these gains.

Age–Period–Cohort analysis showed that mortality from gastric cancer linked to HSI generally declined in China, Japan, Republic of Korea, and Mongolia, with higher risks observed in earlier periods and older birth cohorts. These trends are consistent with the effects of salt-reduction policies, improvements in dietary behaviors, and increased health awareness. All four countries exhibited negative net drift values, reflecting a downward trend after accounting for period and cohort effects. Mongolia had the smallest absolute net drift, indicating a slower decline and underscoring the need for strengthened policies and promotion of low-sodium diets. Notably, the absolute local drift values for the elderly were smaller than the net drift, suggesting that although mortality among older adults decreased from 1990 to 2021, their risk remained elevated compared with other age groups.

Our study identified a negative association between SDI and the disease burden of gastric cancer linked to HSI. From 1990 to 2021, Japan and Republic of Korea saw marked declines in the ASMR and ASDR of HSI-related gastric cancer, while China and Mongolia experienced more gradual reductions—aligning with results from an earlier study on gastric cancer burden in five Asian nations [56]. This underscores that economic growth provides governments with increased resources to roll out salt reduction policies, enhance early gastric cancer detection and management, and invest more in developing novel therapies, which in turn improve gastric cancer treatment outcomes. Furthermore, rising household incomes have boosted refrigerator penetration, reducing reliance on pickling for food storage. This reduction in pickled food consumption has further lessened the disease burden of HSI-related gastric cancer. Economic growth is pivotal to advancing policy rollout, optimizing healthcare provision, and changing population lifestyles. However, cross-country and regional differences demand ongoing focus. Going forward, enhanced international collaboration is needed to attain more equitable gastric cancer control effects, mitigate the burden of HSI-associated gastric cancer, and foster regional and global public health progress.

Decomposition analysis highlighted that epidemiological changes contributed to the mitigation of gastric cancer burden linked to HSI in the four countries, primarily through policy implementation, dietary modifications, and advancements in gastric cancer treatment. In contrast, population aging and growth were the main drivers of the disease burden. Interestingly, in Japan, population aging appeared to act as a mitigating factor for HSI-linked gastric cancer mortality. Japan’s aging society has fostered heightened health awareness among the population, with older adults actively participating in health education programs to understand the risks of HSI, increasingly purchasing low-sodium foods, and reducing their intake of high-sodium products. This shift in health-conscious consumption has contributed to a lower risk of gastric cancer associated with HSI. Since the 1960s, Japan has implemented gastric cancer screening programs for individuals aged over 40, including barium meal and endoscopic examinations [55]. With economic growth and an aging population, healthcare services have increasingly focused on older adults, improving endoscopy coverage and further reducing gastric cancer risk. Additionally, the food environment has been improved in the context of an aging society, with stricter regulation of sodium content in processed foods and mandatory labeling of sodium levels on packaging [57].

The BAPC model forecasts declining trends in the ASMR and ASDR of HSI-related gastric cancer across the four countries in the coming 15 years. Nevertheless, predictions for deaths and DALYs differ: Japan is expected to have fewer deaths and DALYs from HSI-associated gastric cancer, while China and Mongolia will see a more distinct rise. Thus, China and Mongolia should enhance existing policies and scale up low-sodium diet promotion efforts to lower the deaths and DALYs linked to HSI-caused gastric cancer.

Our study found that from 1990 to 2021, the relative burden of gastric cancer linked to HSI declined in China, Japan, Republic of Korea, and Mongolia. This trend is closely associated with efforts to promote healthy dietary habits and increased public health awareness in these countries. With economic growth and the globalization of trade, cultural exchange has intensified, leading to the worldwide dissemination of Chinese, Japanese, and Korean foods [58–60]. In contrast, Mongolian food, known for its distinctive pastoral flavors and abundant meat and dairy products, has received comparatively less international exposure. This study underscores the importance of reducing HSI to mitigate the gastric cancer burden. Countries facing similarly high burdens may benefit from strategies such as adjusting dietary patterns, establishing sodium content standards for processed foods, and implementing salt-reduction policies.

This study has several limitations. First, all data were obtained from the GBD database, and potential biases in data collection and reporting may affect accuracy. Second, the analysis focused solely on the burden of gastric cancer linked to HSI, without accounting for the confounding effects of other risk factors such as smoking and alcohol consumption. Third, due to data limitations, we were unable to further examine disease burden by anatomical site or histological subtype of gastric cancer associated with HSI. Fourth, decomposition analysis indicated that population aging mitigated HSI-linked gastric cancer mortality in Japan; however, as the model does not account for potential confounding from environmental, economic, and social factors, these findings require further validation with additional data.

## Conclusion

In summary, between 1990 and 2021, ASMR and ASDR of gastric cancer linked to HSI decreased in China, Japan, Republic of Korea, and Mongolia, reflecting the effectiveness of salt-reduction policies, gastric cancer screening, and therapeutic improvements. Nevertheless, notable differences persist across gender, age groups, and countries. Policy efforts should focus on men, older adults, and economically disadvantaged regions. Future public health strategies should consider these disparities to further mitigate the gastric cancer burden linked to HSI.

## Supporting information

S1 Fig Age-specific trends in mortality and DALY rates of gastric cancer linked to HSI in 2021. DALY, disability-adjusted life year; HSI, High Sodium Intake.
(TIF)

S2 Fig Trends for the age-specific absolute burden of gastric cancer linked to HSI from 1990 to 2021. (A) Number of deaths among Males; (B) Number of deaths among Females; (C) Number of DALYs among Males; (D) Number of DALYs among Females. HSI, High Sodium Intake; DALYs, disability-adjusted life years.
(TIF)

S3 Fig Decomposition analysis for death and DALYs of gastric cancer linked to HSI in Mongolia. HSI, High Sodium Intake; DALYs, disability-adjusted life years.
(TIF)

S4 Fig Projections for gastric cancer linked to HSI up to 2036 stratified by gender; (A) ASMR and Number of deaths in Korea; (B) ASDR and Number of DALYs in Korea; (C) ASMR and Number of deaths in Mongolia; (D) ASDR and Number of DALYs in Mongolia. ASMR, Age-standardized mortality rate; ASDR, Age-standardized DALYs (disability-adjusted life years) rate; HSI, High Sodium Intake.
(TIF)

S1 Table Joinpoint analysis of the trends of ASMR and ASDR (per 100,000) of gastric cancer linked to HSI from 1990 to 2021 (both sexes, females, males). ASMR, Age-standardized mortality rate; ASDR, Age-standardized DALYs (disability-adjusted life years) rate; HSI, High Sodium Intake; AAPC, Average annual percentage change; APC, annual percentage change; CI, confidence interval. *P < 0.05.
(DOCX)

S2 Table Projections of ASMR, ASDR, number of deaths and DALYs for gastric cancer linked to HSI in China until 2036. ASMR, Age-standardized mortality rate; ASDR, Age-standardized DALYs (disability-adjusted life years) rate; HSI, High Sodium Intake.
(DOCX)

S3 Table Projections of ASMR, ASDR, number of deaths and DALYs for gastric cancer linked to HSI in Japan until 2036. ASMR, Age-standardized mortality rate; ASDR, Age-standardized DALYs (disability-adjusted life years) rate; HSI, High Sodium Intake.
(DOCX)

S4 Table Projections of ASMR, ASDR, number of deaths and DALYs for gastric cancer linked to HSI in Republic of Korea until 2036. ASMR, Age-standardized mortality rate; ASDR, Age-standardized DALYs (disability-adjusted life years) rate; HSI, High Sodium Intake.
(DOCX)

S5 Table Projections of ASMR, ASDR, number of deaths and DALYs for gastric cancer linked to HSI in Mongolia until 2036. ASMR, Age-standardized mortality rate; ASDR, Age-standardized DALYs (disability-adjusted life years) rate; HSI, High Sodium Intake.
(DOCX)
