# Supplementary figures and images for: Trend analysis and projection of gastric cancer burden linked to high sodium intake in China, Japan, Republic of Korea, and Mongolia (1990–2021): A comprehensive assessment based on the 2021 global burden of disease study
Source: PLoS One. 2025 Dec 4;20(12):e0338030. doi: 10.1371/journal.pone.0338030 (PMC12677532; doi:10.1371/journal.pone.0338030)

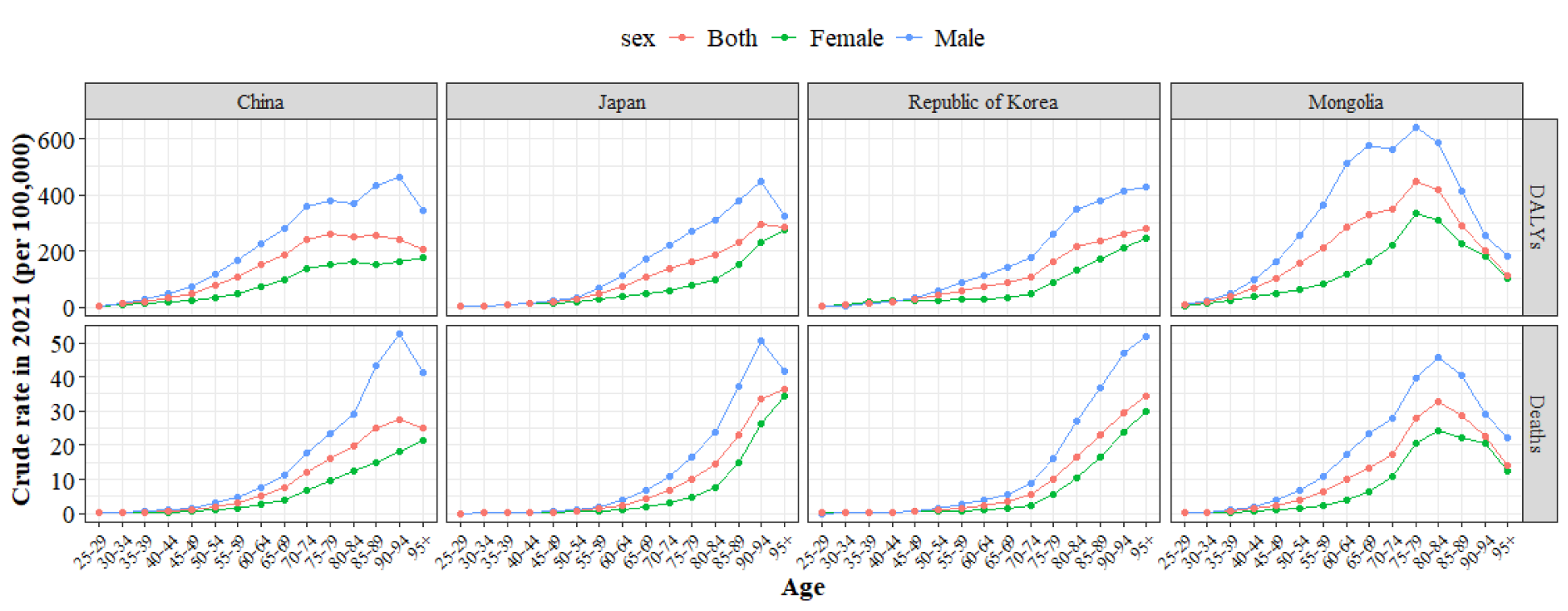

Supplement: S1 Fig — DALY, disability-adjusted life year; HSI, High Sodium Intake. (TIF) [file pone.0338030.s001.tif]

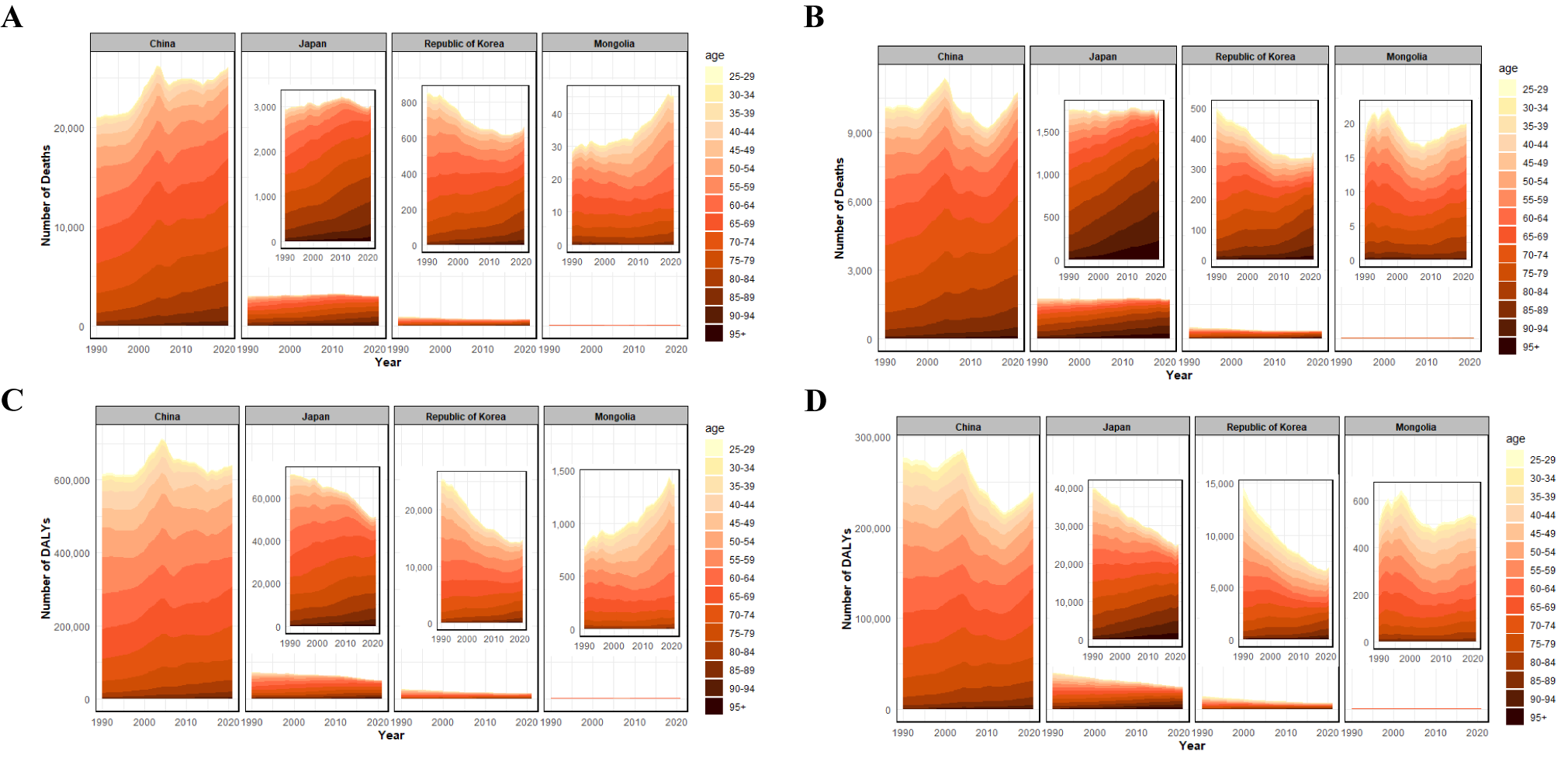

Supplement: S2 Fig — (A) Number of deaths among Males; (B) Number of deaths among Females; (C) Number of DALYs among Males; (D) Number of DALYs among Females. HSI, High Sodium Intake; DALYs, disability-adjusted life years. (TIF) [file pone.0338030.s002.tif]

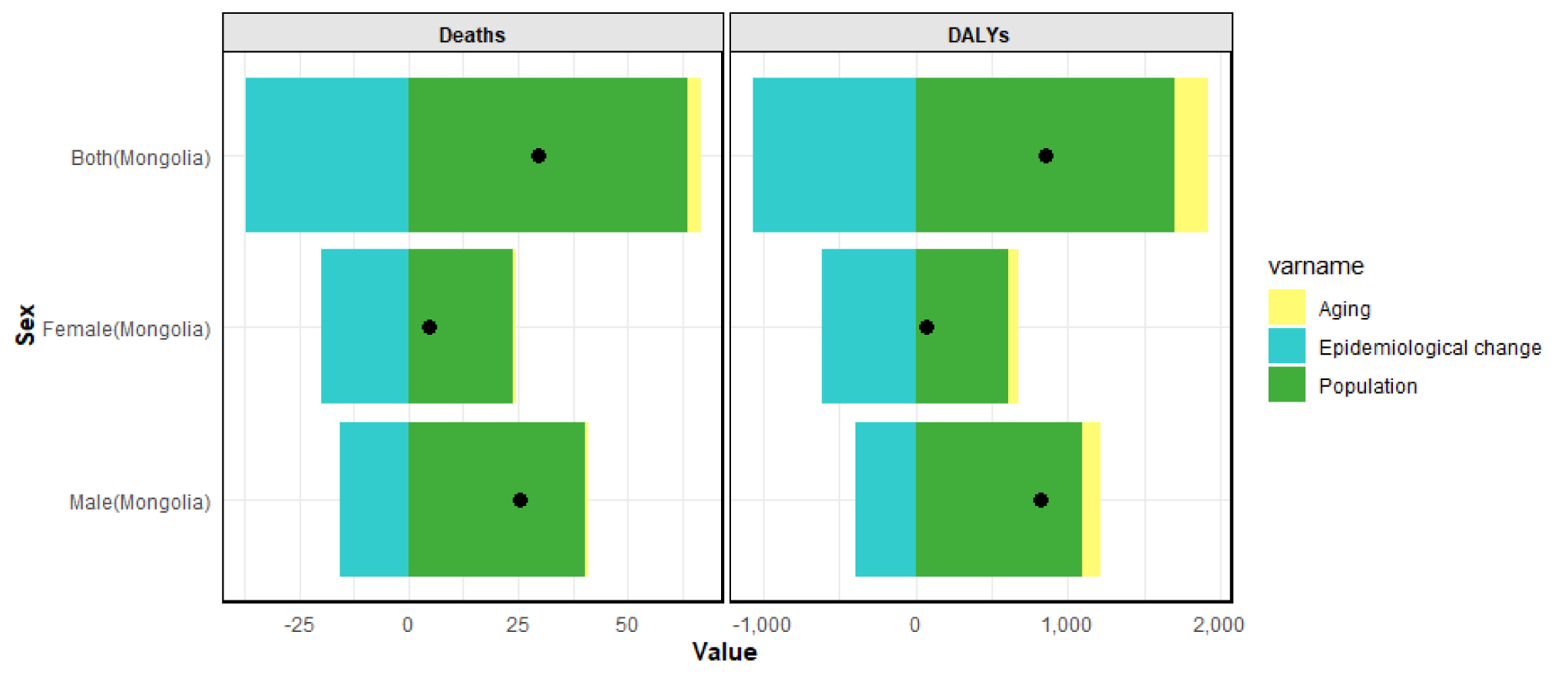

Supplement: S3 Fig — HSI, High Sodium Intake; DALYs, disability-adjusted life years. (TIF) [file pone.0338030.s003.tif]

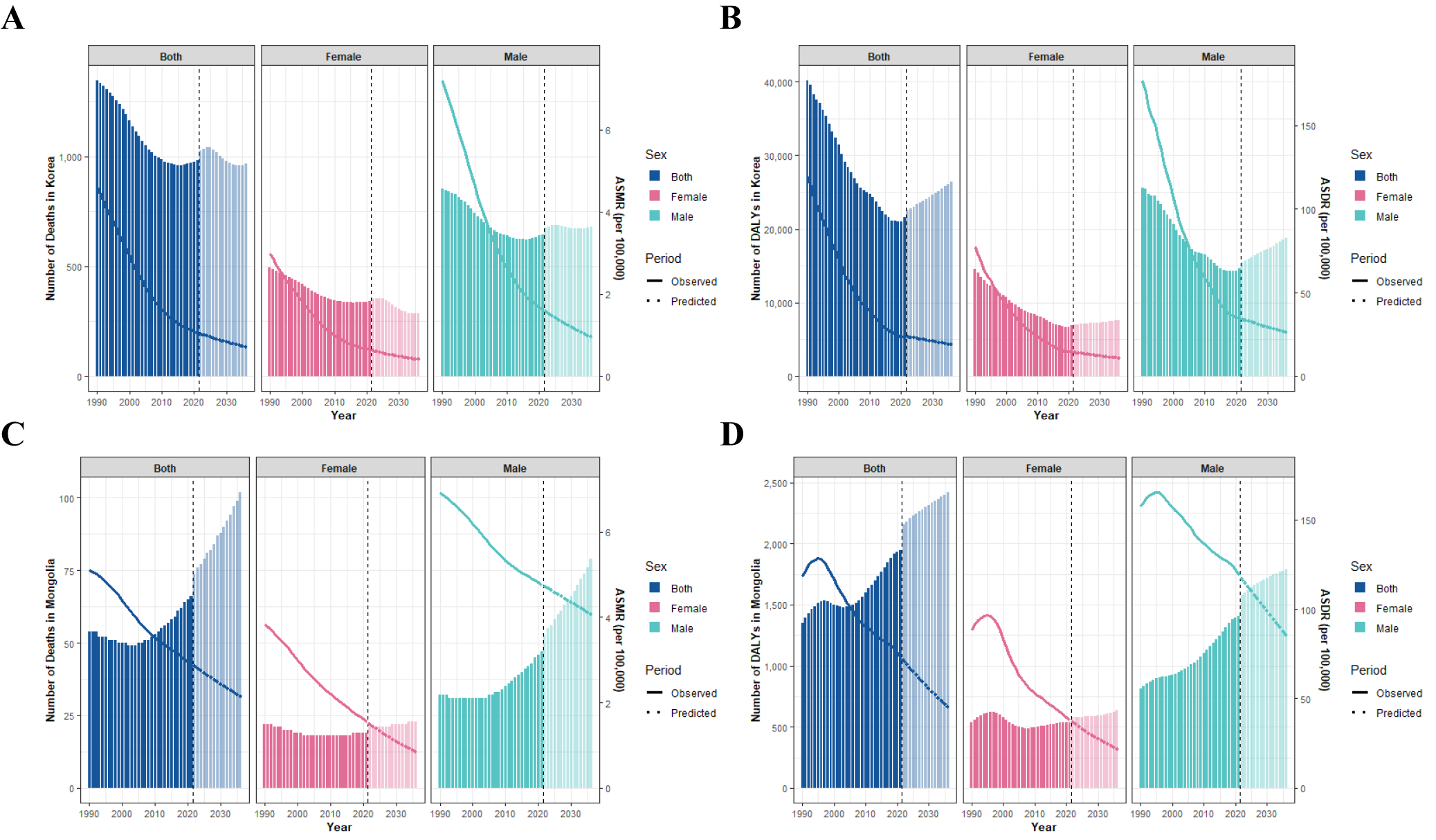

Supplement: S4 Fig — ASMR, Age-standardized mortality rate; ASDR, Age-standardized DALYs (disability-adjusted life years) rate; HSI, High Sodium Intake. (TIF) [file pone.0338030.s004.tif]
