# Supplementary material for: Trend analysis and projection of gastric cancer burden linked to high sodium intake in China, Japan, Republic of Korea, and Mongolia (1990–2021): A comprehensive assessment based on the 2021 global burden of disease study
Source: PLoS One. 2025 Dec 4;20(12):e0338030. doi: 10.1371/journal.pone.0338030 (PMC12677532; doi:10.1371/journal.pone.0338030)
Supplement: S1 Table — ASMR, Age-standardized mortality rate; ASDR, Age-standardized DALYs (disability-adjusted life years) rate; HSI, High Sodium Intake; AAPC, Average annual percentage change; APC, annual percentage change; CI, confidence interval. *P < 0.05. (DOCX) [file pone.0338030.s005.docx]

**S1 Table. Joinpoint analysis of the trends of ASMR and ASDR (per 100,000) of gastric cancer linked to HSI from 1990 to 2021 (both sexes, females, males).**

| **Location** | **Gender** | **Age-standardized mortality rate (per 100,000)** | | | **Age-standardized DALYs rate (per 100,000)** | | |
| --- | --- | --- | --- | --- | --- | --- | --- |
|  |  | **Period** | **APC (95% CI)** | **AAPC (95% CI)** | **Period** | **APC (95% CI)** | **AAPC (95% CI)** |
| China | Both | 1990-1998 | -2.33 (-2.45--2.21)* | -2.45 (-2.61--2.3)* | 1990-1998 | -2.62 (-2.74--2.51)* | -2.76 (-2.91--2.61)* |
| China | Both | 1998-2004 | 0.39 (0.15-0.63)* |  | 1998-2004 | -0.33 (-0.57--0.09)* |  |
| China | Both | 2004-2007 | -6.2 (-7.21--5.17)* |  | 2004-2007 | -6.23 (-7.23--5.23)* |  |
| China | Both | 2007-2010 | -2.79 (-3.84--1.71)* |  | 2007-2010 | -3.46 (-4.49--2.42)* |  |
| China | Both | 2010-2015 | -4.16 (-4.49--3.83)* |  | 2010-2015 | -4.26 (-4.58--3.93)* |  |
| China | Both | 2015-2021 | -1.91 (-2.09--1.73)* |  | 2015-2021 | -1.95 (-2.13--1.77)* |  |
| China | Female | 1990-1998 | -2.66 (-2.81--2.51)* | -2.91 (-3.12--2.7)* | 1990-1998 | -2.95 (-3.15--2.74)* | -3.26 (-3.47--3.04)* |
| China | Female | 1998-2004 | -0.49 (-0.81--0.17)* |  | 1998-2004 | -1.26 (-1.7--0.83)* |  |
| China | Female | 2004-2007 | -7.27 (-8.6--5.92)* |  | 2004-2007 | -7.2 (-9.02--5.35)* |  |
| China | Female | 2007-2010 | -3.97 (-5.36--2.56)* |  | 2007-2014 | -5.18 (-5.5--4.86)* |  |
| China | Female | 2010-2014 | -5.44 (-6.12--4.75)* |  | 2014-2021 | -1.62 (-1.88--1.35)* |  |
| China | Female | 2014-2021 | -1.41 (-1.61--1.21)* |  |  |  |  |
| China | Male | 1990-1998 | -2.2 (-2.36--2.04)* | -2.22 (-2.44--2.01)* | 1990-1998 | -2.49 (-2.63--2.35)* | -2.51 (-2.71--2.32)* |
| China | Male | 1998-2004 | 0.96 (0.62-1.31)* |  | 1998-2004 | 0.16 (-0.15-0.47) |  |
| China | Male | 2004-2007 | -5.67 (-7.08--4.24)* |  | 2004-2007 | -5.62 (-6.89--4.33)* |  |
| China | Male | 2007-2010 | -2.23 (-3.7--0.74)* |  | 2007-2012 | -3.21 (-3.62--2.79)* |  |
| China | Male | 2010-2015 | -3.92 (-4.37--3.46)* |  | 2012-2015 | -4.33 (-5.62--3.02)* |  |
| China | Male | 2015-2021 | -2.2 (-2.45--1.94)* |  | 2015-2021 | -2.11 (-2.34--1.88)* |  |
| Japan | Both | 1990-1999 | -3.01 (-3.13--2.89)* | -3.03 (-3.19--2.87)* | 1990-1999 | -3.33 (-3.45--3.2)* | -3.45 (-3.62--3.29)* |
| Japan | Both | 1999-2002 | -4.02 (-5.32--2.69)* |  | 1999-2002 | -4.37 (-5.71--3.02)* |  |
| Japan | Both | 2002-2012 | -2.66 (-2.79--2.54)* |  | 2002-2012 | -3.11 (-3.23--2.98)* |  |
| Japan | Both | 2012-2019 | -3.68 (-3.9--3.45)* |  | 2012-2019 | -4.29 (-4.53--4.06)* |  |
| Japan | Both | 2019-2021 | -1.25 (-2.6-0.13)* |  | 2019-2021 | -1.38 (-2.78-0.04) |  |
| Japan | Female | 1990-2003 | -3.81 (-3.96--3.66)* | -3.54 (-3.61--3.46)* | 1990-2009 | -4.09 (-4.16--4.02)* | -3.9 (-4.13--3.67)* |
| Japan | Female | 2003-2021 | -3.34 (-3.43--3.25)* |  | 2009-2013 | -3.18 (-4.43--1.91)* |  |
| Japan | Female |  |  |  | 2013-2018 | -4.69 (-5.47--3.91)* |  |
| Japan | Female |  |  |  | 2018-2021 | -2.35 (-3.62--1.06)* |  |
| Japan | Male | 1990-1999 | -2.63 (-2.73--2.52)* | -2.92 (-3.06--2.78)* | 1990-1999 | -3.06 (-3.17--2.95)* | -3.33 (-3.48--3.19)* |
| Japan | Male | 1999-2002 | -3.92 (-5.04--2.78)* |  | 1999-2002 | -4.31 (-5.48--3.13)* |  |
| Japan | Male | 2002-2011 | -2.51 (-2.63--2.38)* |  | 2002-2011 | -2.84 (-2.98--2.71)* |  |
| Japan | Male | 2011-2019 | -3.77 (-3.92--3.62)* |  | 2011-2019 | -4.29 (-4.45--4.13)* |  |
| Japan | Male | 2019-2021 | -1.15 (-2.32-0.04) |  | 2019-2021 | -1.43 (-2.65--0.19)* |  |
| Mongolia | Both | 1990-1992 | 4.52 (0.64-8.54)* | -1.4 (-1.81--1)* | 1990-1992 | 4.28 (1.06-7.6)* | -1.51 (-1.87--1.16)* |
| Mongolia | Both | 1992-1997 | -0.37 (-1.59-0.87)* |  | 1992-1996 | 0.51 (-1.11-2.16) |  |
| Mongolia | Both | 1997-2008 | -2.7 (-3--2.4)* |  | 1996-2008 | -2.8 (-3.02--2.59)* |  |
| Mongolia | Both | 2008-2019 | -1.2 (-1.51--0.88)* |  | 2008-2019 | -1.37 (-1.63--1.11)* |  |
| Mongolia | Both | 2019-2021 | -3.65 (-7.5-0.36) | -1.99 (-2.43--1.55)* | 2019-2021 | -4.14 (-7.26--0.91)* |  |
| Mongolia | Female | 1990-1992 | 6.2 (0.87-11.81)* |  | 1990-1992 | 5.72 (1.27-10.36)* | -2.46 (-2.82--2.1)* |
| Mongolia | Female | 1992-1997 | -0.17 (-1.82-1.51) |  | 1992-1997 | 0.25 (-1.12-1.65) |  |
| Mongolia | Female | 1997-2006 | -4.61 (-5.17--4.04)* |  | 1997-2006 | -5.37 (-5.83--4.9)* |  |
| Mongolia | Female | 2006-2021 | -2.05 (-2.27--1.83)* |  | 2006-2021 | -2.63 (-2.81--2.45)* |  |
| Mongolia | Male | 1990-1992 | 3.88 (-1.24-9.27) | -0.87 (-1.47--0.26)* | 1990-1995 | 1.44 (0.23-2.66)* | -0.95 (-1.43--0.47)* |
| Mongolia | Male | 1992-2004 | -0.87 (-1.22--0.52)* |  | 1995-2014 | -1.43 (-1.61--1.26)* |  |
| Mongolia | Male | 2004-2007 | -3.45 (-8.61-2) |  | 2014-2019 | -0.06 (-1.82-1.72) |  |
| Mongolia | Male | 2007-2021 | -0.97 (-1.22--0.71)* |  | 2019-2021 | -4.47 (-9.75-1.11) |  |
| Republic of Korea | Both | 1990-1999 | -4.12 (-4.32--3.92)* | -4.53 (-4.75--4.31)* | 1990-1997 | -4.81 (-5.06--4.55)* | -5.04 (-5.29--4.78)* |
| Republic of Korea | Both | 1999-2007 | -6.2 (-6.49--5.91)* |  | 1997-2007 | -6.17 (-6.34--6.01)* |  |
| Republic of Korea | Both | 2007-2015 | -4.87 (-5.17--4.57)* |  | 2007-2010 | -4.29 (-6.17--2.38)* |  |
| Republic of Korea | Both | 2015-2019 | -3.43 (-4.55--2.31)* |  | 2010-2015 | -6.06 (-6.64--5.47)* |  |
| Republic of Korea | Both | 2019-2021 | -0.38 (-2.64-1.93) |  | 2015-2019 | -4.01 (-4.95--3.06)* |  |
| Republic of Korea | Both |  |  |  | 2019-2021 | -0.62 (-2.55-1.35) |  |
| Republic of Korea | Female | 1990-1999 | -4.28 (-4.42--4.14)* | -4.68 (-4.83--4.54)* | 1990-1993 | -6.37 (-7.37--5.36)* | -5.16 (-5.43--4.89)* |
| Republic of Korea | Female | 1999-2008 | -6.38 (-6.55--6.22)* |  | 1993-1999 | -5.26 (-5.72--4.79)* |  |
| Republic of Korea | Female | 2008-2014 | -4.91 (-5.26--4.55)* |  | 1999-2005 | -6.25 (-6.7--5.8)* |  |
| Republic of Korea | Female | 2014-2019 | -3.9 (-4.4--3.39)* |  | 2005-2016 | -5.42 (-5.58--5.26)* |  |
| Republic of Korea | Female | 2019-2021 | 0.05 (-1.55-1.67) |  | 2016-2019 | -4.1 (-6.14--2.01)* |  |
| Republic of Korea | Female |  |  |  | 2019-2021 | 0.22 (-1.93-2.4) |  |
| Republic of Korea | Male | 1990-1994 | -3.01 (-3.77--2.23)* | -4.53 (-4.8--4.26)* | 1990-1996 | -4.2 (-4.62--3.77)* | -5.1 (-5.4--4.79)* |
| Republic of Korea | Male | 1994-1999 | -4.62 (-5.39--3.86)* |  | 1996-2007 | -6.26 (-6.45--6.07)* |  |
| Republic of Korea | Male | 1999-2006 | -6.12 (-6.5--5.72)* |  | 2007-2011 | -4.73 (-5.95--3.49)* |  |
| Republic of Korea | Male | 2006-2015 | -5.15 (-5.41--4.9)* |  | 2011-2015 | -6.54 (-7.73--5.34)* |  |
| Republic of Korea | Male | 2015-2019 | -3.51 (-4.71--2.29)* |  | 2015-2019 | -4 (-5.23--2.75)* |  |
| Republic of Korea | Male | 2019-2021 | -0.88 (-3.33-1.64) |  | 2019-2021 | -1.27 (-3.81-1.33) |  |

ASMR, Age-standardized mortality rate; ASDR, Age-standardized DALYs (disability-adjusted life years) rate; HSI, High Sodium Intake; AAPC, Average annual percentage change; APC, annual percentage change; CI, confidence interval. *P < 0.05.
