# Supplementary material for: Trend analysis and projection of gastric cancer burden linked to high sodium intake in China, Japan, Republic of Korea, and Mongolia (1990–2021): A comprehensive assessment based on the 2021 global burden of disease study
Source: PLoS One. 2025 Dec 4;20(12):e0338030. doi: 10.1371/journal.pone.0338030 (PMC12677532; doi:10.1371/journal.pone.0338030)
Supplement: S2 Table — ASMR, Age-standardized mortality rate; ASDR, Age-standardized DALYs (disability-adjusted life years) rate; HSI, High Sodium Intake. (DOCX) [file pone.0338030.s006.docx]

**S2 Table. Projections of ASMR, ASDR, number of deaths and DALYs for gastric cancer linked to HSI in China until 2036**

| Year | Age-standardized mortality rate (per 100,000) | | | Age-standardized DALYs rate (per 100,000) | | | Number of Deaths | | | Number of DALYs | | |
| --- | --- | --- | --- | --- | --- | --- | --- | --- | --- | --- | --- | --- |
|  | Male | Female | Both | Male | Female | Both | Male | Female | Both | Male | Female | Both |
| 2022 | 2.66 | 0.98 | 1.75 | 60.23 | 21.22 | 39.99 | 26871 | 10915 | 37786 | 644180 | 237008 | 881188 |
| 2023 | 2.61 | 0.97 | 1.72 | 58.94 | 20.87 | 39.17 | 27113 | 11134 | 38247 | 644688 | 239525 | 884213 |
| 2024 | 2.56 | 0.96 | 1.69 | 57.68 | 20.53 | 38.36 | 27370 | 11356 | 38726 | 645479 | 242302 | 887781 |
| 2025 | 2.52 | 0.95 | 1.66 | 56.45 | 20.19 | 37.58 | 27635 | 11591 | 39226 | 646390 | 245229 | 891619 |
| 2026 | 2.47 | 0.93 | 1.62 | 55.26 | 19.86 | 36.82 | 27905 | 11836 | 39741 | 647358 | 248169 | 895527 |
| 2027 | 2.42 | 0.92 | 1.59 | 54.12 | 19.53 | 36.08 | 28152 | 12087 | 40239 | 648023 | 250993 | 899016 |
| 2028 | 2.37 | 0.91 | 1.56 | 53.02 | 19.21 | 35.36 | 28401 | 12360 | 40761 | 648622 | 253877 | 902499 |
| 2029 | 2.33 | 0.90 | 1.54 | 51.96 | 18.90 | 34.67 | 28691 | 12669 | 41360 | 649694 | 257131 | 906825 |
| 2030 | 2.28 | 0.89 | 1.51 | 50.93 | 18.60 | 34.00 | 29023 | 13011 | 42034 | 651378 | 260790 | 912168 |
| 2031 | 2.24 | 0.87 | 1.48 | 49.94 | 18.31 | 33.36 | 29399 | 13377 | 42776 | 653910 | 264833 | 918743 |
| 2032 | 2.19 | 0.86 | 1.45 | 49.01 | 18.03 | 32.74 | 29797 | 13764 | 43561 | 657242 | 269236 | 926478 |
| 2033 | 2.15 | 0.85 | 1.43 | 48.12 | 17.75 | 32.15 | 30243 | 14190 | 44433 | 661723 | 274217 | 935940 |
| 2034 | 2.11 | 0.84 | 1.40 | 47.28 | 17.49 | 31.59 | 30771 | 14675 | 45446 | 667710 | 280086 | 947796 |
| 2035 | 2.07 | 0.83 | 1.38 | 46.47 | 17.24 | 31.04 | 31382 | 15218 | 46600 | 675201 | 286897 | 962098 |
| 2036 | 2.04 | 0.82 | 1.35 | 45.70 | 16.99 | 30.52 | 32072 | 15813 | 47885 | 684355 | 294645 | 979000 |

ASMR, Age-standardized mortality rate; ASDR, Age-standardized DALYs (disability-adjusted life years) rate; HSI, High Sodium Intake.
