# Supplementary material for: Trend analysis and projection of gastric cancer burden linked to high sodium intake in China, Japan, Republic of Korea, and Mongolia (1990–2021): A comprehensive assessment based on the 2021 global burden of disease study
Source: PLoS One. 2025 Dec 4;20(12):e0338030. doi: 10.1371/journal.pone.0338030 (PMC12677532; doi:10.1371/journal.pone.0338030)
Supplement: S3 Table — ASMR, Age-standardized mortality rate; ASDR, Age-standardized DALYs (disability-adjusted life years) rate; HSI, High Sodium Intake. (DOCX) [file pone.0338030.s007.docx]

**S3 Table. Projections of ASMR, ASDR, number of deaths and DALYs for gastric cancer linked to HSI in Japan until 2036.**

| Year | Age-standardized mortality rate (per 100,000) | | | Age-standardized DALYs rate (per 100,000) | | | Number of Deaths | | | Number of DALYs | | |
| --- | --- | --- | --- | --- | --- | --- | --- | --- | --- | --- | --- | --- |
|  | Male | Female | Both | Male | Female | Both | Male | Female | Both | Male | Female | Both |
| 2022 | 1.59 | 0.61 | 1.05 | 32.23 | 12.69 | 21.71 | 2887 | 1671 | 4558 | 50175 | 24134 | 74309 |
| 2023 | 1.54 | 0.59 | 1.02 | 31.65 | 12.34 | 21.24 | 2855 | 1657 | 4512 | 49892 | 23807 | 73699 |
| 2024 | 1.49 | 0.58 | 0.99 | 31.09 | 11.99 | 20.77 | 2819 | 1643 | 4462 | 49611 | 23481 | 73092 |
| 2025 | 1.45 | 0.56 | 0.96 | 30.58 | 11.65 | 20.32 | 2775 | 1626 | 4401 | 49371 | 23143 | 72514 |
| 2026 | 1.40 | 0.54 | 0.93 | 30.07 | 11.33 | 19.89 | 2722 | 1603 | 4325 | 49148 | 22797 | 71945 |
| 2027 | 1.36 | 0.53 | 0.90 | 29.48 | 11.01 | 19.44 | 2661 | 1575 | 4236 | 48722 | 22425 | 71147 |
| 2028 | 1.31 | 0.51 | 0.87 | 28.89 | 10.70 | 18.99 | 2598 | 1546 | 4144 | 48250 | 22039 | 70289 |
| 2029 | 1.27 | 0.49 | 0.85 | 28.35 | 10.40 | 18.55 | 2537 | 1517 | 4054 | 47796 | 21656 | 69452 |
| 2030 | 1.22 | 0.48 | 0.82 | 27.85 | 10.10 | 18.13 | 2479 | 1488 | 3967 | 47425 | 21277 | 68702 |
| 2031 | 1.18 | 0.47 | 0.79 | 27.38 | 9.82 | 17.73 | 2420 | 1460 | 3880 | 47119 | 20908 | 68027 |
| 2032 | 1.14 | 0.45 | 0.77 | 26.85 | 9.54 | 17.33 | 2359 | 1432 | 3791 | 46646 | 20530 | 67176 |
| 2033 | 1.10 | 0.44 | 0.74 | 26.32 | 9.27 | 16.93 | 2299 | 1404 | 3703 | 46121 | 20145 | 66266 |
| 2034 | 1.06 | 0.42 | 0.72 | 25.82 | 9.01 | 16.53 | 2241 | 1378 | 3619 | 45613 | 19769 | 65382 |
| 2035 | 1.02 | 0.41 | 0.69 | 25.37 | 8.75 | 16.15 | 2185 | 1354 | 3539 | 45206 | 19400 | 64606 |
| 2036 | 0.99 | 0.40 | 0.67 | 24.95 | 8.50 | 15.80 | 2129 | 1329 | 3458 | 44898 | 19041 | 63939 |

ASMR, Age-standardized mortality rate; ASDR, Age-standardized DALYs (disability-adjusted life years) rate; HSI, High Sodium Intake.
