# Supplementary material for: Trend analysis and projection of gastric cancer burden linked to high sodium intake in China, Japan, Republic of Korea, and Mongolia (1990–2021): A comprehensive assessment based on the 2021 global burden of disease study
Source: PLoS One. 2025 Dec 4;20(12):e0338030. doi: 10.1371/journal.pone.0338030 (PMC12677532; doi:10.1371/journal.pone.0338030)
Supplement: S4 Table — ASMR, Age-standardized mortality rate; ASDR, Age-standardized DALYs (disability-adjusted life years) rate; HSI, High Sodium Intake. (DOCX) [file pone.0338030.s008.docx]

**S4 Table. Projections of ASMR, ASDR, number of deaths and DALYs for gastric cancer linked to HSI in Republic of Korea until 2036.**

| Year | Age-standardized mortality rate (per 100,000) | | | Age-standardized DALYs rate (per 100,000) | | | Number of Deaths | |  | Number of DALYs | |  |
| --- | --- | --- | --- | --- | --- | --- | --- | --- | --- | --- | --- | --- |
|  | Male | Female | Both | Male | Female | Both | Male | Female | Both | Male | Female | Both |
| 2022 | 1.57 | 0.62 | 1.04 | 34.23 | 14.35 | 23.62 | 675 | 353 | 1028 | 15441 | 7051 | 22492 |
| 2023 | 1.52 | 0.6 | 1.01 | 33.62 | 14.06 | 23.28 | 681 | 354 | 1035 | 15691 | 7085 | 22776 |
| 2024 | 1.47 | 0.58 | 0.98 | 33.02 | 13.79 | 22.93 | 687 | 355 | 1042 | 15938 | 7121 | 23059 |
| 2025 | 1.42 | 0.57 | 0.96 | 32.4 | 13.52 | 22.59 | 689 | 352 | 1041 | 16169 | 7156 | 23325 |
| 2026 | 1.37 | 0.55 | 0.93 | 31.78 | 13.25 | 22.24 | 687 | 345 | 1032 | 16397 | 7186 | 23583 |
| 2027 | 1.33 | 0.53 | 0.91 | 31.18 | 12.98 | 21.89 | 683 | 336 | 1019 | 16620 | 7211 | 23831 |
| 2028 | 1.28 | 0.52 | 0.88 | 30.59 | 12.73 | 21.56 | 679 | 325 | 1004 | 16848 | 7238 | 24086 |
| 2029 | 1.23 | 0.5 | 0.86 | 30 | 12.48 | 21.23 | 676 | 315 | 991 | 17080 | 7273 | 24353 |
| 2030 | 1.19 | 0.49 | 0.83 | 29.41 | 12.23 | 20.9 | 674 | 306 | 980 | 17304 | 7312 | 24616 |
| 2031 | 1.15 | 0.47 | 0.81 | 28.84 | 11.99 | 20.57 | 672 | 299 | 971 | 17537 | 7355 | 24892 |
| 2032 | 1.11 | 0.46 | 0.79 | 28.28 | 11.76 | 20.24 | 671 | 293 | 964 | 17774 | 7399 | 25173 |
| 2033 | 1.07 | 0.44 | 0.77 | 27.74 | 11.53 | 19.93 | 671 | 288 | 959 | 18017 | 7451 | 25468 |
| 2034 | 1.03 | 0.43 | 0.75 | 27.19 | 11.3 | 19.61 | 673 | 286 | 959 | 18260 | 7512 | 25772 |
| 2035 | 1 | 0.42 | 0.73 | 26.63 | 11.08 | 19.29 | 676 | 285 | 961 | 18495 | 7582 | 26077 |
| 2036 | 0.96 | 0.41 | 0.71 | 26.09 | 10.86 | 18.97 | 680 | 287 | 967 | 18745 | 7658 | 26403 |

ASMR, Age-standardized mortality rate; ASDR, Age-standardized DALYs (disability-adjusted life years) rate; HSI, High Sodium Intake
