# Supplementary material for: Trend analysis and projection of gastric cancer burden linked to high sodium intake in China, Japan, Republic of Korea, and Mongolia (1990–2021): A comprehensive assessment based on the 2021 global burden of disease study
Source: PLoS One. 2025 Dec 4;20(12):e0338030. doi: 10.1371/journal.pone.0338030 (PMC12677532; doi:10.1371/journal.pone.0338030)
Supplement: S5 Table — ASMR, Age-standardized mortality rate; ASDR, Age-standardized DALYs (disability-adjusted life years) rate; HSI, High Sodium Intake. (DOCX) [file pone.0338030.s009.docx]

**S5 Table. Projections of ASMR, ASDR, number of deaths and DALYs for gastric cancer linked to HSI in Mongolia until 2036.**

| Year | Age-standardized mortality rate (per 100,000) | | | Age-standardized DALYs rate (per 100,000) | | | Number of Deaths | | | Number of DALYs | | |
| --- | --- | --- | --- | --- | --- | --- | --- | --- | --- | --- | --- | --- |
|  | Male | Female | Both | Male | Female | Both | Male | Female | Both | Male | Female | Both |
| 2022 | 4.72 | 1.49 | 2.85 | 117.10 | 36.86 | 71.33 | 53 | 21 | 74 | 1581 | 580 | 2161 |
| 2023 | 4.67 | 1.44 | 2.80 | 114.78 | 35.51 | 69.20 | 55 | 21 | 76 | 1602 | 581 | 2183 |
| 2024 | 4.62 | 1.39 | 2.74 | 112.47 | 34.21 | 67.11 | 56 | 21 | 77 | 1623 | 582 | 2205 |
| 2025 | 4.58 | 1.33 | 2.69 | 110.18 | 32.95 | 65.08 | 58 | 21 | 79 | 1643 | 583 | 2226 |
| 2026 | 4.53 | 1.28 | 2.64 | 107.90 | 31.74 | 63.09 | 60 | 21 | 81 | 1661 | 584 | 2245 |
| 2027 | 4.49 | 1.23 | 2.58 | 105.64 | 30.57 | 61.16 | 61 | 21 | 82 | 1678 | 585 | 2263 |
| 2028 | 4.44 | 1.18 | 2.53 | 103.38 | 29.45 | 59.26 | 63 | 21 | 84 | 1693 | 587 | 2280 |
| 2029 | 4.40 | 1.14 | 2.48 | 101.12 | 28.37 | 57.41 | 65 | 22 | 87 | 1708 | 590 | 2298 |
| 2030 | 4.35 | 1.09 | 2.43 | 98.86 | 27.32 | 55.59 | 66 | 22 | 88 | 1722 | 593 | 2315 |
| 2031 | 4.30 | 1.05 | 2.38 | 96.62 | 26.32 | 53.81 | 68 | 22 | 90 | 1735 | 597 | 2332 |
| 2032 | 4.25 | 1.00 | 2.33 | 94.38 | 25.35 | 52.08 | 70 | 22 | 92 | 1746 | 602 | 2348 |
| 2033 | 4.21 | 0.96 | 2.28 | 92.14 | 24.42 | 50.38 | 72 | 22 | 94 | 1757 | 608 | 2365 |
| 2034 | 4.16 | 0.92 | 2.24 | 89.91 | 23.53 | 48.72 | 74 | 23 | 97 | 1767 | 616 | 2383 |
| 2035 | 4.11 | 0.88 | 2.19 | 87.68 | 22.67 | 47.08 | 76 | 23 | 99 | 1777 | 625 | 2402 |
| 2036 | 4.06 | 0.85 | 2.14 | 85.47 | 21.84 | 45.49 | 79 | 23 | 102 | 1787 | 636 | 2423 |

ASMR, Age-standardized mortality rate; ASDR, Age-standardized DALYs (disability-adjusted life years) rate; HSI, High Sodium Intake
